# Control of *Mycobacterium avium* subsp. *paratuberculosis* load within infected bovine monocyte-derived macrophages is associated with host genetics

**DOI:** 10.3389/fimmu.2023.1042638

**Published:** 2023-02-22

**Authors:** Gerard Badia-Bringué, María Canive, Marta Alonso-Hearn

**Affiliations:** ^1^ Department of Animal Health, NEIKER-Basque Institute for Agricultural Research and Development, Basque Research and Technology Alliance (BRTA), Derio, Bizkaia, Spain; ^2^ Doctoral Program in Molecular Biology and Biomedicine, Universidad del País Vasco/Euskal Herriko Unibertsitatea (UPV/EHU), Leioa, Bizkaia, Spain

**Keywords:** paratuberculosis, mycobacterial growth inhibition assay (MGIA), host resistance, macrophages, innate immune response, phagocytosis, immunoprofiling

## Abstract

The genetic loci influencing individual resistance to *Mycobacterium avium* subsp. *paratuberculosis* (MAP) infection are still largely unknown. In the current study, we searched for genetic loci associated with resistance to MAP infection by evaluating the performance of monocyte-derived macrophages (MDMs) isolated from the peripheral blood of 75 healthy Holsteins cows and infected *ex vivo* with MAP. Bacterial load (log colony-forming units, log CFUs) within MDMs was quantified at 2 h and 7 days p. i. using a BACTEC MGIT 960 instrument. In addition, the expression levels of some genes with important roles in the innate immune response including epiregulin (EREG), complement component C3 (C3), galectin-9 (Gal9), and nitric oxide (NO^-^) were measured in the supernatant of the infected cells. DNA from peripheral blood samples of the animals included in the study was isolated and genotyped with the EuroG MD bead Chip (44,779 single nucleotide-polymorphisms, SNPs). Linear mixed models were used to calculate the heritability (*h^2^*) estimates for each indicator of MDM performance, MAP load within MDMs and EREG, C3, Gal9, and NO^-^expression. After performing a genome-wide association study, the only phenotypes that showed SNPs with a significant association were the bacterial load within MDMs at 2 h (*h^2^* = 0. 87) and 7 days (*h^2^* = 0.83) p.i. A total of 6 SNPs, 5 candidate genes, and one microRNA on the *Bos taurus* chromosomes BTA2, BTA17, BTA18, and BTA21 were associated with MAP load at 2 h p.i. Overlap was seen in two SNPs associated with the log CFUs at 2 h and 7 d p.i. The identified SNPs had negative regression coefficients, and were, therefore, associated with a low bacterial load within MDMs. Some of the identified SNPs were located within QTLs previously associated with longevity, reproductive, and udder health traits. Some of the identified candidate genes; *Oxysterol Binding Protein Like 6, Cysteine and Serine Rich Nuclear Protein 3, and the Coiled-Coil Domain Containing 92* regulate cellular cholesterol trafficking and efflux, apoptosis, and interferon production, respectively. Taken together, our results define a heritable and distinct immunogenetic profile in MAP-infected macrophages designed to limit bacterial load early after infection.

## 1 Introduction

Bovine paratuberculosis (PTB) or Johne´s disease (JD) is a chronic enteritis of domestic and wild ruminants caused by *Mycobacterium avium* subsp. *paratuberculosis* (MAP). PTB is a major problem for animal health that compromises animal welfare and causes important economic losses to the dairy industry (1). In Europe and North America, PTB is endemic in dairy cattle, with herd prevalence estimates higher than 50% (2). PTB-associated losses include increased susceptibility to other diseases, increased somatic cell counts, increased incidence of clinical mastitis, reduced fertility, reduced milk production, costs of testing, and involuntary culling of cows (3). Animals are infected early in life through the fecal-oral route but clinical onset appears when animals are 18 months or older (4). Once ingested, MAP reaches the jejune and ileum and crosses the intestinal mucosa by binding to fibronectin β1 receptors present on M cells located in Peyer’s patches (5, 6). In the submucosa, MAP is phagocytized by sub-epithelial macrophages. Within infected macrophages, MAP has developed several survival mechanisms such as preventing the maturation and acidification of the phagosome and its fusion with the lysosome and preventing the presentation of antigens to the immune system (7). MAP has a zoonotic potential and it has been detected in samples of patients with Crohn´s disease (CD), ulcerative colitis, and idiopathic inflammatory bowel disease (IBD)-associated colorectal cancer (8–11). Inactivated vaccines against MAP interfere with diagnostic tests for bovine tuberculosis and therefore are not allowed in many countries. One solution to control this disease is the identification of genetic loci determining variation in immune-related traits that could be used to select PTB-resistant animals.

PTB is a multifactorial disease that arises as the result of the interaction of genetic, environmental, and microbial factors which affect the various disease outcomes. According to their extension in the intestine, cellular infiltrate, and amount of MAP; PTB-associated lesions were classified as focal, multifocal, and diffuse (diffuse paucibacillary or lymphoplasmacytic, diffuse intermediate, and diffuse multibacillary or histiocytic) (12, 13). Our research group has explored very recently the associations between PTB-associated pathology and host genetics (14). Overall, a total of 380 SNPs were found associated (FDR ≤ 0.05; *P* ≤ 5 10^-7^) with ante-mortem (serum ELISA) and post-mortem (tissue PCR and culture and histopathology) PTB diagnostic definitions in a common set of Spanish Holstein cattle (N = 983) using whole-genome sequence data (WGS) data (14–16). However, we were unable to identify SNPs associated with resistance to MAP infection.

Reductionist approaches investigate a host biological subsystem such as a key cellular function whose performance is the criterion for the classification of the population (17). Recently, a strong effect of host genetics on the *in vitro* nitric oxide (NO^-^) production of monocyte-derived macrophages (MDMs) in response to two common bacterial pathogens of dairy cattle, *Escherichia coli* and *Staphylococcus aureus*, has been described (18). Macrophages are important host defense cells against MAP infection. Since MAP-infected macrophages’ performance can be better analyzed under a controlled environment, the probability of identifying resistant animals is much higher using *ex vivo* macrophage models in comparison to holistic approaches based on field studies. In addition, it is generally hypothesized that mycobacteria growth in macrophages *in vitro* predicts the *in vivo* risk of disease or infection (19, 20). Moreover, the direct mycobacteria growth inhibition assay (MGIA) has demonstrated cross-species potential (21). Recently, we have demonstrated that MAP-infected MDMs constitute a very useful model of infection to validate the functional impact of specific expression quantitative trait loci (cis-eQTLs) potentially implicated in susceptibility to MAP infection (22). Cis-eQTLs are genetic variants typically located in gene regulatory regions that alter gene expression in an allele-specific manner. Cis-eQTLs are functional links between genomic variants, gene expression, and ultimately phenotype. In the current study, we hypothesize that MAP-infected MDMs might help to identify resistant individuals that respond stronger than others to MAP infection and, as result, reduce the intracellular MAP burden significantly.

**Figure 1:**
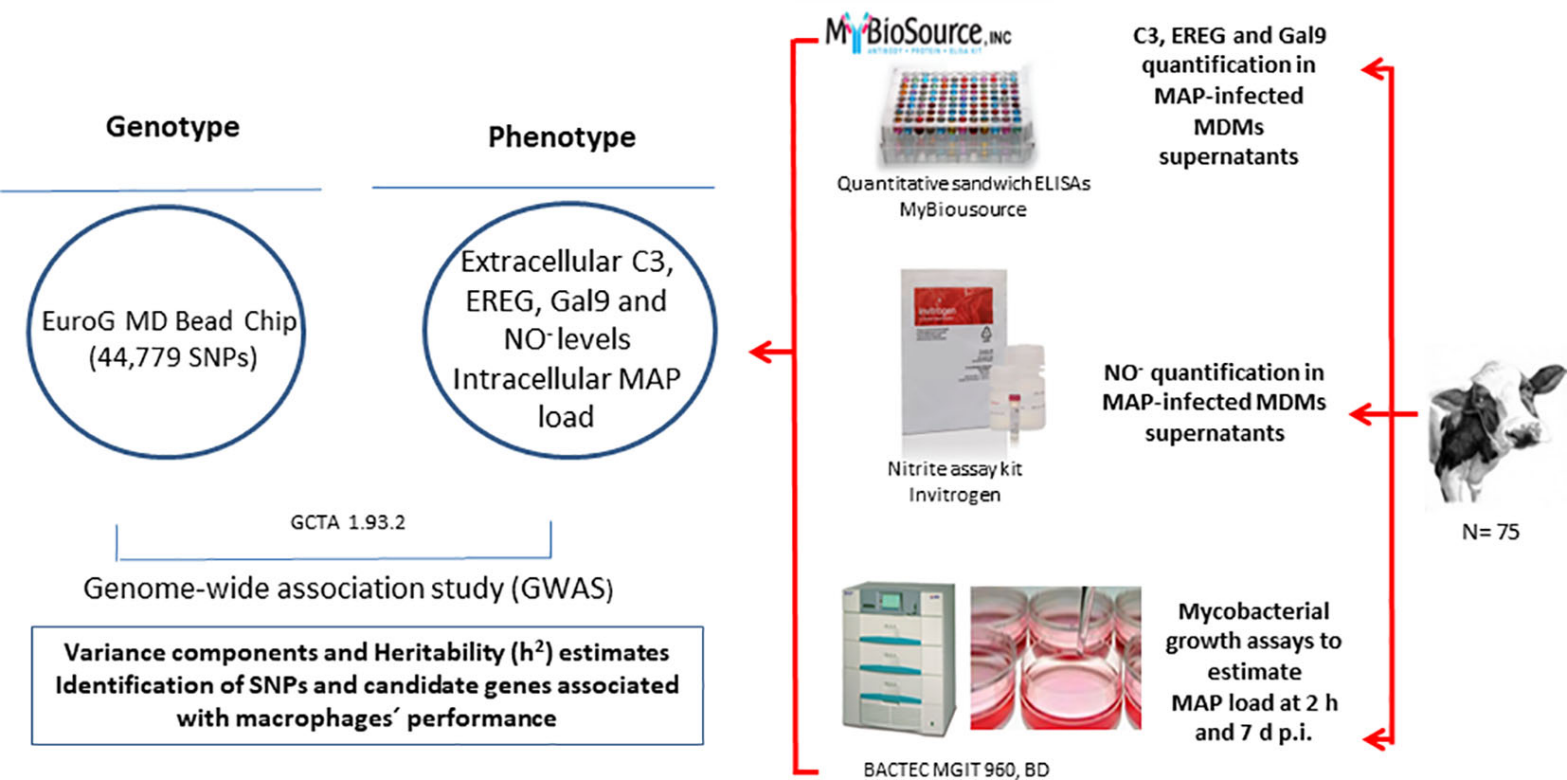
Study design. The approach starts from the measures of MAP load within MAP infected-MDMs and the C3, EREG, Gal9, and NO^-^ expression levels in the supernatants of MAP-infected-MDMs. These phenotypes are combined with the genotypes of the animals in a GWAS study for the calculation of variance components and heritability *(h^2^*) estimates and the identification of associated SNPs and candidate genes.

The measure of indicators of MDMs’ performance in response to MAP infection is another strategy for the identification of resistant cattle. To date, however, no serum biomarkers have been associated with resistance to MAP infection. In our previous study (22), the integration of gene expression data (RNA-Seq) and genotypes (54,609 SNPs per animal) from a cohort of cows naturally exposed to MAP allowed the identification of 192 cis-eQTLs associated with the expression of 145 genes in peripheral blood samples. Four of these cis-eQTLs regulated the expression of several genes with important roles in the innate immune response; the complement component C3 (C3), epiregulin (EREG), galectin-9 (Gal9), and nitric oxide synthase (NOS1). In the current study, we tested whether the measurement of MAP load within infected MDMs and the expression in the supernatants of MAP-infected MDMs of several genes with important roles in the innate immune response could be used as correlates of MDMs performance. Our objective was to identify genetic loci associated with resistance to MAP infection using *ex vivo* MAP-infected MDMs from Spanish Holstein cows. For this purpose, we performed a genome-wide association study (GWAS) to identify SNPs and candidate genes associated with the MAP load within infected MDMs and with the expression of Gal9, ERG, C3, and NO^-^ in the supernatants of infected MDMs. Since there is evidence that some allelic variants may contribute to resistance to multiple pathogens, the identified SNPs and candidate genes were compared with QTLs and candidate genes for other bovine diseases and health and reproductive traits. In addition, the candidate genes identified in our study were compared with human candidate genes previously identified for CD, IBD, and colorectal cancer. The workflow of the study is presented in **Figure 1**.

## 2 Materials and methods

### 2.1 Ethical statement

Animals used in this study were not submitted to any *in vivo* experimentation. Blood sampling was conducted with the approval of the Ethics Committee of Animal Experimentation of NEIKER (OEBA-2022-010), following current legal regulations and in particular, according to pertinent Basque (Basque Government Decree 454/1994), Spanish (Spanish Government Law 32/2007/7 and Royal decree 53/2013/1), and European (Community Council Animal Welfare Guidelines 2010/63/EU) regulations.

### 2.2 Animals and blood samples

The reference population consisted of 75 Spanish Holstein cows from a single farm located in the Basque Country. In 2022, only five cows from this herd had an ELISA positive result and all the animals had a negative fecal PCR result. Only adult cows (2 years or older, 4.5 years mean age) were included in the study. All the animals included in this study were registered with the Spanish Federation of Holstein cattle (CONAFE; www.conafe.com.) and had negative fecal PCR results when blood samples were collected and in subsequent annual samplings. In addition, the animals used in this study were not diagnosed with any other pathogen. Blood samples were collected in groups of 16 per sampling day. The validation population consisted of 16 cows from the same farm.

### 2.3 Mycobacterial growth inhibition assays

MGIA were performed as previously described (22–24). Briefly, fifteen milliliters of peripheral blood were drawn from the tail vein of healthy Holstein cows into heparinized Vacutainer tubes (Becton, Dickinson and Company, Sparks, MD, USA) and diluted 1:2 in Hanks balanced salt solution (HBSS). Leucosep tubes were filled with 15 ml of Ficoll-Paque (1.084 g/cm^3^) (GE Healthcare, Uppsala, Sweden) and centrifuged at 1,000 rpm for 30 seconds at room temperature. Subsequently, the diluted blood was overlaid on the top of the Ficoll-Paque and centrifuged at 800 g for 15 minutes at room temperature. The plasma layer was removed and the cell interphase containing peripheral blood mononuclear cells (PBMCs) was collected and transferred to a clean tube. PBMCs were washed twice in HBSS and centrifuged at 400 g for 10 minutes to remove platelets. Supernatants were aspirated and the purified PBMCs were resuspended in RPMI-1640 supplemented with 20 mM L-glutamine, 10% heat-inactivated bovine serum (Lonza, Spain), 100 U ml-1 penicillin G, and 100 mg ml-1 streptomycin sulfate (Lonza, Spain). PBMCs were cultured at a concentration of 1 x 10^6^ cells/ml into 24-well plates and incubated at 37°C in a humidified 5% CO_2_ incubator for 2 h. Non-adherent cells were removed by washing, and adherent cells were incubated in fresh medium for 7 days at 37°C to allow differentiation to MDMs. Differentiated MDMs were inoculated in triplicate with a single-cell suspension of MAP K10 strain at a multiplicity of infection (MOI) of 10:1 (bacteria:cells). After 2 h, the supernatant was removed, and the cells were washed twice with HBSS to remove extracellular bacteria. Infected MDMs were lysed at this time (2 h p. i.) or were cultured at 37°C for 7 days in fresh medium. At each time point, the supernatant was aspirated and infected MDMs were lysed by vigorous pipetting with 0.5 ml of 0.1% Triton X-100 (Sigma-Aldrich) in sterile water for 10 min.

### 2.4 Assessment of viable intracellular MAP using the BACTEC MGIT 960 system.

MGIA assessment using the BACTEC MGIT 960 system was performed as previously described (23–25). Briefly, supplemented Mycobacteria Growth Indicator tubes (MGIT) (Becton, Dickinson, and Company, Sparks, MD) were inoculated with 0.5 ml of the cell lysates. The concentration of the initial suspension of the MAP K10 strain used to inoculate the MDMs was validated in the BACTEC MGIT 960. The tubes were incubated at 37 ± 2°C for up to 42 days in a BACTEC MGIT 960 instrument (Becton, Dickinson, and Company). The earliest instrumental indicator of positivity (i.e., time to detection [TTD]) for each tube was recorded. The predicted number of bacteria in each positive tube was calculated using standard curves that relate TTD (in days) to the estimated log CFUs (25). For the standard curves, a ten-fold dilution series of a MAP K10 strain cellular suspension (McFarland= 1) were prepared and the TTDs of 100 µl of each dilution in the BACTEC MGIT 960 system were detected. MAP load (log CFUs) were plotted versus TTDs and the mathematical equation was used to determine the estimated log CFUs for each sample. The log CFU ratios were calculated by dividing the estimated log CFUs at day 7 by that at 2 h p. i. MAP survival indexes were calculated according to Martinez et al. (26), and Price et al. (27), as the square root CFU ratio percentage at 7 days p. i. with respect to 2 h p. i. Lower values of the MAP survival index reflect higher resistance to the infection.

### 2.5 NO^-^ quantification

NO^-^ levels were measured in the supernatants collected from the MAP-infected MDMs after 2 h of infection with the Measure-iT ™ High-Sensitivity Nitrite Assay Kit (Invitrogen, Paisley, UK) according to the manufacturer's instructions. The assay has an optimal range of 20–500 pmol nitrite, making it up to 50 times more sensitive than colorimetric methods utilizing the Griess reagent. Nitrates are analyzed after quantitative conversion to nitrites through enzymatic reduction; used in this manner, the assay provides effective quantitation of NO**^-^**. Briefly, the supernatants from the MAP-infected MDMs after 2 h of infection (40 µl) were brought to a volume of 50 µl by adding 10 µl of H_2_O and placed into 96-well black plates in triplicate. Subsequently, 100 µl of quantitation reagent were added and mixed by pipetting. The plate was incubated for 10 minutes at room temperature and 5 µl of quantitation developer were added to each well and gently mixed by pipetting. Right after quantitation developer addition, fluorescence was measured using a multimodal microplate reader Synergy™ HTX (Biotek, Winooski, Vermont, US) at 365/450 nm (ex/em). A standard curve was prepared with the Measure-iT™ nitrite quantitation reagent concentrate included in the kit (0, 2.75, 5.5, 11, 22, 33, 44, 55 µM). Assays were conducted in duplicate in a final volume of 110 µl by adding 100 µl of quantification agent, fluorescence was measured, and plotted versus picomoles of NO^-^. The equation was used to determine the NO^-^ concentration for each sample.

### 2.6 Quantification of EREG, Gal9, and C3 protein levels

The EREG, Gal9, and C3 expression was assessed in the supernatants of MAP-infected MDMs collected at 7 days p. i. using quantitative sandwich ELISAs according to the manufacturer’s instructions (MyBioSource, San Diego, US). The sensitivity of the EREG, Gal9, and C3 ELISA kits is 5 pg/ml (detection range, 31.2-1.000 pg/ml), 0.1 ng/ml (detection range, 0.625-20 ng/ml), and 2 µg/ml (detection range, 15.6 μg/ml-500 μg/ml), respectively. Briefly, standards and samples (50 µl) were added in duplicate into a Microelisa Stripplate provided with each kit. One hundred microliters of horseradish peroxidase-conjugated antibody were added to each well. After incubation for 60 min at 37°C in the dark, the plate was washed four times with 350 µl of wash solution and incubated with 50 µl of 3, 3′, 5, 5′-Tetramethylbenzidine for 15 min at 37°C in the dark. After adding 50 µl of stop solution into each well, the OD values were measured in an ELISA reader at 450 nm (Thermo Scientific Multiskan, US). We average the duplicate readings for each standard and sample and subtract the average OD of the blank. A standard curve was generated by plotting the mean OD values of each standard on the vertical axis and the corresponding concentration on the horizontal axis. The levels of EREG, Gal9, and C3 in each sample were interpolated from the standard curve. The correlations between the intracellular MAP load within MDMs at 2 h and 7 d p. i., and the quantification of EREG, Gal9, and C3 protein levels were analyzed using the Spearman’s rank correlation coefficient (ρ) implemented in *R 4.1.2*. considering a coefficient with a *P*-value less than or equal to 0.05 as significant.

### 2.7 Genotyping

Peripheral blood samples were collected in EDTA tubes and DNA was extracted using the QIAmp DNA Blood Mini Kit according to the manufacturer’s instructions (Qiagen, Hilden, Germany). Purified genomic DNA was genotyped with the EuroG MD Bead Chip at the molecular genetic laboratory service of the Spanish Federation of Holstein Cattle (CONAFE). The InfiniumTM iScan software (Illumina, San Diego, CA) was used for allele assignation. All the SNPs passed a call rate > 0.80. After filtering out SNPs with minimum allele frequency (MAF)< 0.01, the final marker set included 44,779 SNPs.

### 2.8 Variance components and heritability (*h^2^*) estimates

The intracellular MAP load at 2 h and 7 d p. i. and the expression of NO^-^, EREG, Gal9, and C3 were the quantitative phenotypes analyzed. The variance components and *h^2^* explained by all the SNPs were calculated using the genome-wide complex trait analysis (*GCTA*) software 1.93.2, according to the following formula


$$h^{2}=\frac{\sigma_{G}^{2}\;}{\sigma_{G}^{2}+\;\sigma_{e}^{2}}$$

where $\sigma_{G}^{2}$ Is the variance explained by all the SNPs and $\sigma_{e}^{2}$ is the residual variance (28).

### 2.9 Genome-wide association study

Genotypes and the quantitative phenotypes were analyzed using the mixed linear model association analysis of the *GCTA* 1.93.2 software. Briefly, the model is *y = a + bx + g + e*, where *y* is the phenotype, *a* is the mean term, *b* is the additive effect (fixed effect) of the candidate SNP to be tested for association, *x* is the SNP genotype indicator variable coded as 0, 1 or 2, *g* is the polygenic effect (random effect) assumed to be distributed as N~(0 $\sigma_{G}^{2}$ ),and *e* is the residual assumed to be distributed as N~(0 $\sigma_{e}^{2}$ ). Age was included as a covariate in the analysis. To account for multiple testing, a 5% genome-wide false discovery rate (*FDR*) was used (29). The inflation factor (*λ*) and quantile-quantile plots were used to compare the observed distributions of –log (*P*-values) to the expected distribution under the no-association model. *λ* values close to 1 suggest appropriate adjustment for potential substructure and *λ* > 1.2 suggest population stratification. The regression coefficients (*b-*values) were calculated using the *GCTA 1.93.2* software.

### 2.10 Identification of SNPs and candidate genes

The EuroG MD Bead chip is based on the University of Maryland UMD 3.1 *Bos taurus* reference genome; hence, the coordinates of the GWAS-identified SNPs were converted to the ARS-UCD1.2 genome by using *liftOver* software (https://genome.ucsc.edu). The location of the identified SNPs (e.g. upstream or downstream of a transcript, in the coding sequence, in non-coding RNA, in regulatory regions) in the ARS-UCD1.2 genome was determined using the Ensembl Variant Effect predictor (VEP). None of the identified SNPs were within 500,000 base pairs of each other or on linkage disequilibrium. The candidate genes located within 50,000 base pairs to each side of the SNPs were identified using *Ensembl* (https://www.ensembl.org). The function of all the identified genes was searched in GeneCards (http://www.genecards.org) by searching their gene symbol. Since there is evidence that some allelic variants may contribute to resistance to multiple pathogens, the identified SNPs and candidate genes were compared with QTLs and candidate genes previously associated with other bovine diseases, longevity, and reproductive and health traits (http://www.animalgenome.org). In addition, the identified candidate genes were also compared with human candidate genes previously identified for CD, IBD, and colorectal cancer (http://www.ebi.ac.uk/gwas).

### 2.11 Genomic estimated breeding values for MAP survival indexes within MDMs and validation of the genomic predictions

gEBVs for MAP survival indexes within MDMs were calculated for each animal in the reference population using the *gBLUP* model of *GCTA 1.93.2* (30). Subsequently, gEBVs for MAP survival indexes within MDMs were predicted in a validation population. PBMCs were isolated from animals with high (N = 8) and low (N = 8) gEBVs, differentiated to MDMs, infected *ex vivo* with MAP, and at 2 h and 7 d p. i. the intracellular load was quantified in the BACTEC MGIT system as described in point 2.3. An unpaired student t-test with the Welch-Satterthwaite correction was used to compare the experimental MAP survival indexes calculated for the animals with high and low gEBVs (GraphPad Prism 8, San Diego, CA, US). Differences were considered significant when *P*-values were ≤ 0.05. Correlations between the gEBVs and experimental MAP survival indexes within infected MDMs were calculated with the Pearson correlation implemented in *R 4.1.2*.

## 3 Results

### 3.1 Functional phenotyping of bovine MDMs in response to MAP infection

**Figure 2:**
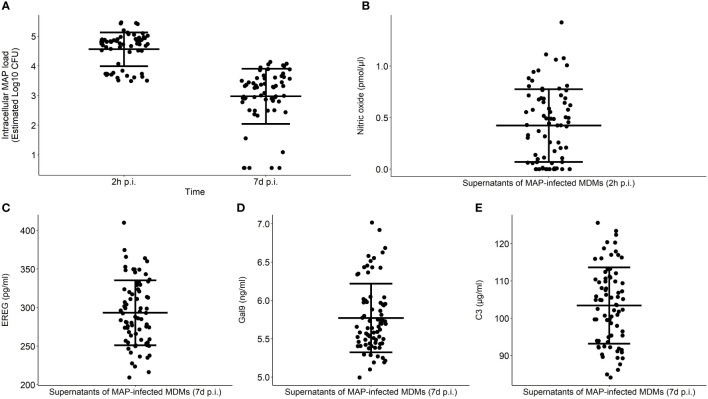
Functional phenotyping of bovine MDMs in response to MAP infection. MDMs performance was determined by measuring the intracellular MAP load at 2 h and 7 d p. i. **(A)** and extracellular NO^-^ **(B)**, EREG **(C)**, Gal9 **(D)**, and C3 production **(E)** in response to MAP infection. Extracellular NO^-^ levels were measured in the supernatants of MAP-infected MDMs at 2 h p. i. while EREG, Gal9, and C3 production were tested at 7 d p. i. Each dot represents one individual response, the middle line represents the average in each group, and the error bars represent the standard deviation from the mean in each group.

As indicators of bovine MDMs performance, intracellular MAP load within MDMs at 2 h and 7 d p. i. and NO^−^, EREG, Gal9, and C3 secretion were measured in MAP-infected MDMs isolated from 75 cows (**Figure 2**). The NO^-^ secretion was measured in the supernatants of MAP-infected MDMs at 2 h p.i. while EREG, Gal9, and C3 were measured at 7 d p. i. To estimate the technical reproducibility of each phenotype, the technical coefficients of variation (CVs) were calculated for each phenotype. The technical CV calculated using the duplicate measures within one experiment for C3, EREG, Gal9, NO^-^, and MAP load with macrophages at 2 h and 7 d p. i. were 6.47% (0% - 17.54%), 4.76% (0% - 10.92%), 4.08% (0% - 9.48%), 14.07% (0.89% - 76.69%), 2.00% (0% - 7.12%), and 7.11% (0% - 33.89%), respectively. To estimate the variation within the population, the CV were calculated for the measures of each phenotype within the population. The CV of Gal9, C3, EREG, NO^-^, and MAP load within MDMs at 2 h and 7 d p. i. were 7, 9, 14, 41, 12, and 31%, respectively. The biological reproducibility of the MAP load within macrophages at 2 h and 7 d p. i. was previously assessed by calculating the CVs of biological triplicates within three different experiments; 9.40 and 10.72%, respectively (24). The biological CV considers the precision of the quantification of MAP load within macrophages to correctly characterize the cow or the fidelity of the reading of the phenotype. The correlations between the six phenotypes were analyzed using Spearman’s rank correlation (**Table 1**). The strongest positive correlation (ρ = 0.80) was found between the intracellular MAP load at 2 h and 7 d p. i., while the highest negative correlation (ρ = - 0.64) was observed between the secretion of NO^-^ measured at 2 h p. i. and the intracellular MAP load at 7 d p. i. The NO^-^ measured at 2 h p. i. also correlated negatively with the intracellular MAP load at 2 h p. i. (ρ = - 0.29). The secretion of C3 was positively correlated with the MAP intracellular load at 2 h and 7 d p.i.; ρ = 0.34 and ρ= 0.48, respectively. On the other hand, negative correlations were found between NO^-^ and Gal9, and NO^-^ and EREG expression; ρ = - 0.24 and ρ = - 0.35, respectively. Positive correlations were found between Gal9 land EREG, and Gal9 and C3 expression; ρ = 0.28 and ρ = 0.44, respectively.

**Table 1:** Spearman’s rank correlation coefficient (**ρ**) between the intracellular MAP load within MDMs at 2 h and 7 d p. i., and the extracellular Gal9, EREG, C3, and NO^-^ levels.

Spearman’s ρ	Gal9	EREG	C3	NO^-^	MAP load	MAP load
	(ng/ml)	(pg/ml)	(µg/ml)	(pg/µl)	(log CFU 2h)	(log CFU 7d)
Gal9 (ng/ml)	1	**0.28**	**0.44**	**-0.24**	0.001	0.16
EREG (pg/ml)		1	0.09	**-0.35**	-0.23	0.03
C3 (µg/ml)			1	-0.17	**0.34**	**0.48**
NO^-^ (pg/µl)				1	**-0.29**	**-0.64**
MAP load (log CFU 2h)					1	**0.80**
MAP load (log CFU 7d)						1

The significant results (*P*-value ≤ 0.05) are presented in bold.

### 3.2 Variance components and heritability (*h^2^*) estimates

The intracellular MAP load at 2 h and 7 d p. i. and the extracellular NO^-^, EREG, Gal9, and C3 levels were the quantitative phenotypes analyzed. The variance components, additive genetic variance ( $\sigma_{G}^{2}$ ) and residual variance ( $\sigma_{e}^{2}$ ), and *h^2^* explained by all the SNPs were calculated using the genome-wide complex trait analysis (*GCTA*) software 1.93.2. **Table 2** summarizes the variance components and *h^2^* estimates for each phenotype. The highest *h^2^* estimates were obtained for the MAP load within MDMs at 2 h (*h^2^ = *0.87) and 7 days p. i. *(h^2^* = 0.83). The estimated *h^2^* for C3 (*h^2^ = *0.78), EREG (*h^2^* = 0.74), NO^-^ (*h^2^* = 0.44) and Gal9 (*h^2^* = 0.13) levels were also calculated. While the *h^2^* estimates for intracellular MAP load, C3 (*h^2^ = *0.78), and EREG (*h^2^* = 0.74) were high; NO^-^ (*h^2^* = 0.44) and Gal9 (*h^2^* = 0.13) estimates were moderate to low.

**Table 2:** Variance components and h^2^ estimates for the intracellular MAP load within MDMs at 2 h and 7 d p. i., and the extracellular Gal9, EREG, C3, and NO^-^ levels.

Phenotype	Additive genetic variance ( $\sigma_{G}^{2}$ )	Residual variance ( $\sigma_{e}^{2}$ )	Heritability (h^2^)
MAP load (logCFU 2h)	0.289112	0.040153	0.878053
MAP load (logCFU 7d)	0.742780	0.144624	0.837025
C3	112.929703	30.072801	0.789704
EREG	1,275.659780	434.419796	0.745965
NO^-^	0.056510	0.071659	0.440901
Gal9	0.027960	0.174846	0.137864

### 3.3 GWAS

**Figure 3:**
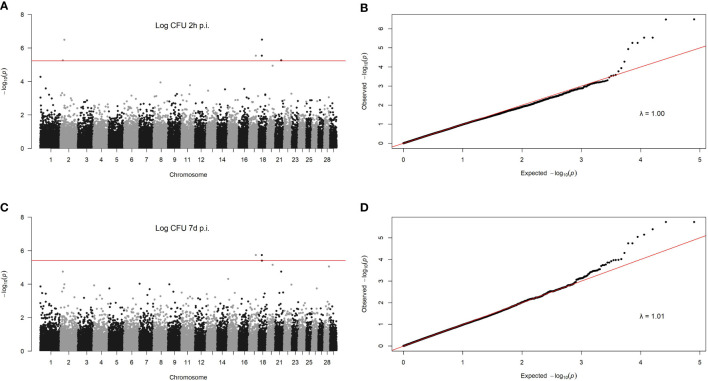
GWAS for MAP load within infected MDMs. Manhattan plots showing –log10 (P-values) of association between every single SNP and intracelllar MAP load within MDMs at 2 h **(A)** and 7 d p. i. **(B)** and 7 d p. i. **(C)**. Each dot represents the result from the test association for a single SNP. The chromosomes´ locationof the SNPs is indicated on the x-axis. The horizontal red line represents the genome-wide significance threshold –log_10_ (P_FDR_). Quantile-quantileplots comparing the observed distribution of –log (P-values) for the intracellular MAP load within MDMs at 2 h **(D)** to the expected values under the null hypothesis.

To identify associations between genetic variants and phenotypes, a GWAS was performed for each phenotype. Only the bacterial load within MDMs at 2 h and 7 d p. i. showed SNPs with a statistically significant association. Six SNPs surpassed the *P_FDR_* correction for the MAP load at 2 h p. i. and two for the MAP load at 7 d p. i. Overlap was seen in two SNPs associated with MAP load within MDMs at 2 h and 7 d p. i. These two SNPs were located on BTA17 and BTA18. Manhattan plots showing –log_10_ (*P*-values) of association between each SNP and MAP load within MDMs at 2 h and 7 d p. i. are presented in **Figures 3A, B**, respectively. Quantile-quantile plots comparing the observed distribution of –log (*P*-values) to the expected values under the null hypothesis for the MAP load within MDMs at 2 h and 7 d p. i. are presented in **Figures 3C, D**, respectively. The plots showed a distribution close to the expected line; λ_median_= 1.00 and λ_median_= 1.01 for MAP load at 2 h and 7 d p. i., respectively. This indicates that significant values were not overestimated due to population stratification or cryptic relatedness.

### 3.4 SNPs associated with MAP load within MDMs and candidate genes identification

The SNPs surpassing the significance threshold (*P_FDR_* ≤ 0.05), *P*-values, and candidate genes are presented in **Table 3**. The six identified SNPs were located on BTA2, BTA17, BTA18, and BTA21. Three of the SNPs were in intergenic regions, two in introns, and one is a downstream variant. The most genome-wide significantly associated SNP (*P* = 3.28 × 10^-7^) was located on BTA2. The regression coefficients (*b*-values) of the six identified SNPs were all negative suggesting that all the SNPs were associated with a low bacterial load within MDMs. None of the six SNPs identified by GWAS was in strong linkage disequilibrium. A total of five candidate genes and one miRNA were identified within 50,000 bp upstream and downstream of the identified SNPs including the *Cysteine and Serine Rich Nuclear Protein 3* (*CSRNP3*), *U6 spliceosomal RNA* (*U6*), *Oxysterol Binding Protein Like 6* (*OSBPL6)*, *Coiled-Coil Domain Containing 92* (*CCDC92*), ENSBTAG00000042858, and bta-mir-2365. Two of the six identified SNPs were in intergenic regions and showed no evidence of a candidate gene within 100,000 bp distance.

**Table 3:** SNPs surpassing the significance threshold (*P_FDR_* ≤ 0.05) for evidence of an association with MAP load within infected MDMs at 2 h and 7 d p. i.

Phenotype	SNP ID	BTA^1^	*P*-value	Annotation	Regression coefficient (*b*-value)	Genes in QTL^2^
MAP load 2h p.i. (log CFUs)	1	2	3,283E-07	Intron	-0.627772	*CSRNP3, U6*
	2	2	5,567E-06	Intron	-0.821413	*OSBPL6,ENSBTAG00000042858*
	3	17	2,944E-06	Downstream	-0.918562	*CCDC92*
	4	18	3,236E-07	Intergenic	-0.688961	
	5	18	2,944E-06	Intergenic	-0.918562	
	6	21	5,567E-06	Intergenic	-0.821413	bta-mir-2365
MAP load 7d p. i. (log CFUs)	3	17	1,860E-06	Downstream	-1.535665	*CCDC92*
	4	18	1,860E-06	Intergenic	-1.535665	

^1^ Chromosome location, ^2^ Candidate genes located within 50,000 bp upstream and dowsntream of each significant SNP. FDR: false discovery rate

### 3.5 Comparison of the identified SNPs and candidate genes to previously reported QTLs and candidate genes

The SNPs and candidate genes that were identified in the current study had not been associated with bovine PTB, tuberculosis, or clinical mastitis before. By examining the available cattle QTL database (**Table 4**), we observed that two of the identified SNPs were located on BTA18 within QTLs previously associated with the somatic cell score (QTL:3556), length of productive life (QTL:3555), and reproductive traits such as stillbirth (QTL:11362), birth index (QTL: 30537), and dystocia (QTL:2707) (31–34). In addition, the SNP located on BTA21 was located within QTLs associated with IgG levels (QTL:66212), somatic cell score (QTL:5446 and QTL:10190), and calving ease (QTL:11126) (35–38). Finally, the SNP on BTA17 was located within a QTL associated with calving ease (36). The identified candidate genes have not been associated with PTB or with other diseases in cattle or in other animal species.

**Table 4:** Overlapping of the identified SNPs with QTLs associated with other health and reproductive traits.

Phenotype	SNP ID	IgG level	Somatic cell score	Length of productive life	Stillbirth	Birth index	Dystocia	Calving ease
MAP load 2 h p.i. (logCFUs)	3							QTL:11051
	4		QTL:3556	QTL:3555	QTL:11362	QTL:30537	QTL:2707	
	5		QTL:3556	QTL:3555	QTL:11362	QTL:30537	QTL:2707	
	6	QTL:66212	QTL:5446, QTL:10190					QTL:11126
MAP load 7d p.i. (logCFUs)	3							QTL:11051
	4		QTL:3556	QTL:3555	QTL:11362	QTL:30537	QTL:2707	

### 3.6 Validation of the genomic predictions

**Figure 4:**
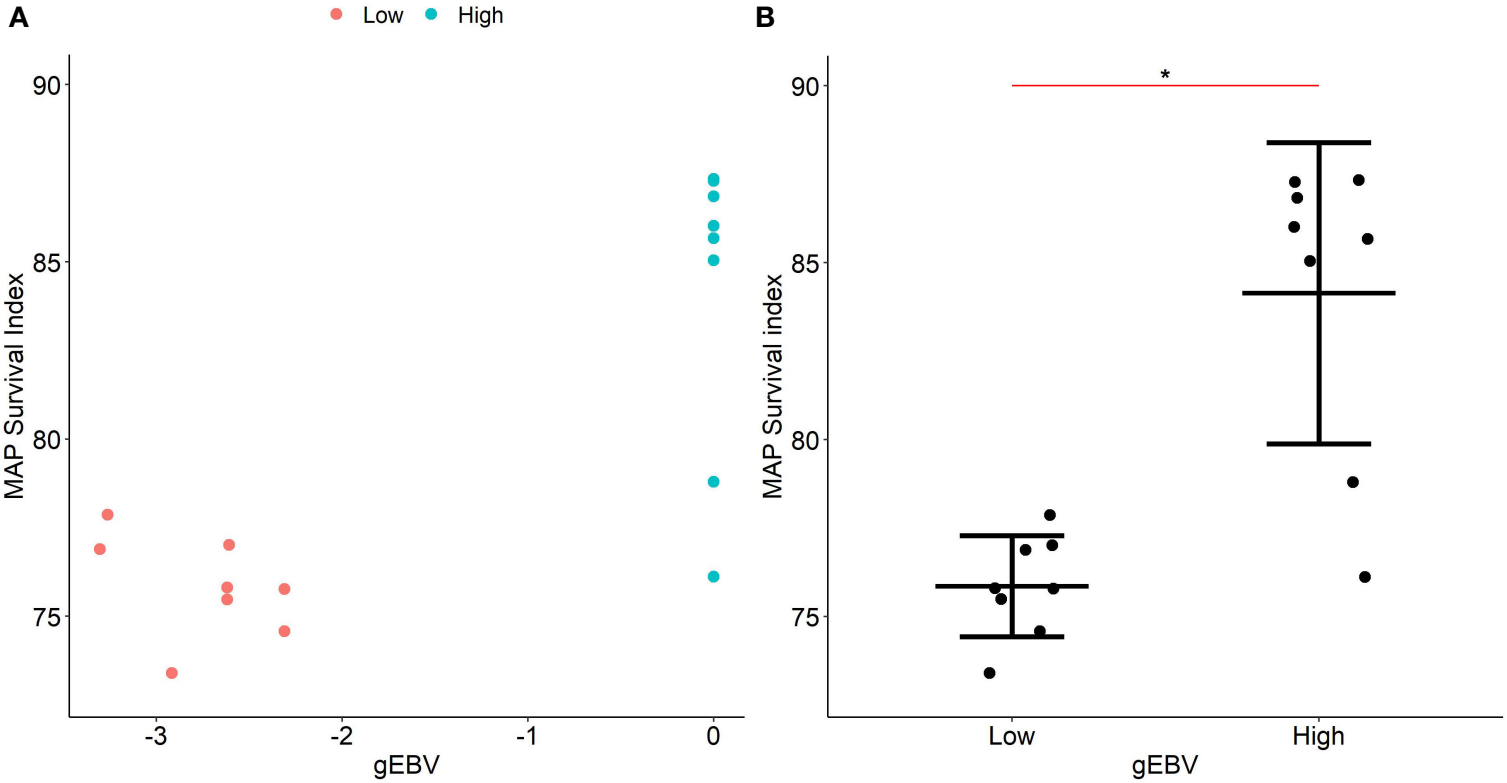
Validation of the gEBVs for MAP survival indexes within infected MDMs. **(A)**. Scatter plots showing the distribution of the gEBVs for MAP survival within infected MDMs for the validation population. gEBVs for MAP survival indexes within MDMs were calculated for each animal in the study population using a *gBLUP* model and the genomic predictions were validated in a validation population consisting of animals with high (N= 8) and low (N= 8) gEBVs. **(B)**. Experimental MAP survival indexes within MDMs from the validation population. MDMs from animals with high (N= 8) and low (N= 8) gEBVs were isolated, infected with MAP for 2 h and 7 d p. i., and the MAP survival indexes were calculated. Differences in the MAP survival indexes between animals with high and low gEBVs were statistically significant (*P<* 0.0006). In the validation population, the correlations between the experimental MAP survival indexes within MDMs and the gEBVs were positive (Pearson *r* = 0.78, *P* = 0.0003). The asterisk indicates statistically significant differences.

The *gBLUP* model (30) was used to estimate gEBVs of each individual in the study population based on the effect of the six SNPs with evidence of association with the survival indexes of MAP within MDMs and their genomic relationships. Subsequently, the six SNPs with evidence of association with the phenotype were used to predict the gEBVs of the validation population. For the validation of the genomic predictions, 16 animals with high (N = 8) and low (N = 8) gEBVs were selected. The distribution of the gEBVs is presented in **Figure 4A**. MDMs of the selected animals were isolated and infected *ex vivo* with MAP as previously described in Materials and Methods. Intracellular bacterial load was quantified at 2 h and 7 d p. i. and the experimental MAP survival indexes were calculated for the animals with low (mean = 75.7) and high gEBVs (mean = 84.1). There were significant differences in the killing capacity of macrophages from cows with low and high gEBVs (*P-*value = 0.0006) and the data demonstrated a tighter grouping of the macrophages from cows with low gEBVs having superior killing capacity (**Figure 4B**). Pearson correlation coefficient between the gEBVs and experimental MAP survival indexes was positive (0.78) (*P*-value = 0.0003). These results demonstrated a significant effect of the six identified SNPs on MAP survival within macrophages and confirmed the existence of a strong genetic effect on macrophages’ performance in response to MAP infection.

## 4 Discussion

Macrophages are important antigen presenter cells that participate in the destruction of pathogens by producing antimicrobial components after phagocytosis (39–44). Some animals might be able to induce a strong early innate immune response and limit MAP load within macrophages better than others. A considerable proportion of this inter-individual variability might be genetic (16). Defining the relevant phenotype is the main challenge in identifying resistant animals and the genetic profile of resistance against MAP infection. Since *ex vivo* assays measure the capacity of a cell population to respond to a challenge, functional testing represents the best way to evaluate resistance and vaccine protection (45). MGIA assays using bovine MDMs were previously used to assess macrophages´ s capacity to clear MAP strains isolated from different hosts (23, 24), to test novel therapeutic approaches for the treatment of MAP-infected animals (46), and to test vaccines’ efficacy and protection (47). Our study is the first in using a quantitative MGIA as an unbiased measure of the bovine MDMs´ ability to control MAP survival *in vitro* and as a correlate of resistance to MAP infection. Since the MGIA models MAP infection, it reveals immune parameters induced by *in vivo* infection and contributes to understanding the control of intracellular MAP load. In addition, evidence exists that the ability of the macrophages to produce an antimicrobial profile early after infection can cause differences in disease susceptibility or resistance in connection to their cellular responses (48–50). Previously, next-generation RNA (RNA-Seq) sequencing was used to study the transcriptomic response to MAP infection of the macrophages from cows that were naturally infected and identified as positive for JD (JD (+); n = 22) or negative for JD (healthy/resistant, JD (−); n = 28) (51). In addition to identifying genetic variants from RNA-seq data, SNP variants were also identified using the Bovine SNP50 DNA chip. Significant variants from JD (+/-) macrophages were identified by a genome-wide association study in a case-control approach and revealed two novel eQTLs on BTA4 and 11 (*P*< 5 × 10-^7^). This study was conducted using MDMs from cattle that were naturally infected with MAP. In our study, however, we used MDMs obtained from cattle that were negative by PCR for the detection of MAP DNA in fecal samples before blood collection and in subsequent annual samplings. Our study is the first in using a quantitative MGIA as an unbiased measure of the bovine MDMs´ ability to control MAP load and as a correlate of resistance to MAP infection. In our previous study (22), the integration of RNA-Seq data and genotypes from a cohort of cows naturally exposed to MAP allowed the identification of four cis-eQTLs regulating the expression of genes with important roles in the induction of the innate immune response early after infection; the *C3*, *EREG*, *Gal9*, and *NOS1* genes. As shown in **Supplementary Figure 1**, none of the animals with the highest levels of expression of these genes showed diffuse lesions, which suggested that the measure of the levels of expression of these genes might be used to evaluate macrophages performance and for the identification of cattle able to limit MAP infection. The goal of the current study was to identify genetic loci associated with resistance to MAP infection by combining genotypes and phenotypes including the intracellular MAP load within infected MDMs and the extracellular expression of NO^-^, Gal9, C3, and EREG.

NOS1 belongs to the family of NO^-^ synthases which synthesize NO^-^ from L-arginine. NO^-^ is one of the major macrophage-killing mechanisms (52). It was previously demonstrated that MAP interferes with NO^-^ synthase expression early after infection (53). EREG belongs to the *Epidermal Growth factor* (EGF) family together with the Heparin Binding EGF-like Growth factor (HBEGF); acting both as mitogenic stimulators *via* binding to EGF receptors (EGFRs). EREG overexpression leads to downstream signaling events including Mitogen-Activated Protein Kinase 8 (MAPK8) phosphorylation and activation which results in apoptosis induction. Using RNA-Seq transcriptomic profiling, we previously detected down-regulation of EREG in peripheral blood and ileocecal valve from cows with diffuse lesions versus controls which may favor MAP survival (54). Gal9 is an S-type lectin with antimicrobial activity in infected macrophages by causing macrophage activation, Interleukin 1ß (IL1ß) secretion, and pathogen clearance (55, 56). Higher C3 levels may allow increased opsonophagocytosis and effective bacterial clearance of several microorganisms including mycobacteria (47, 57). In our study, negative correlations were observed between the extracellular NO^-^ and the MAP load within infected MDMs at 2 h and 7 d p. i. The correlations between EREG and Gal9 and intracellular MAP load within MDMs were not statistically significant. On the other hand, positive correlations were found between MAP load within macrophages and C3 expression at 2 h and 7 d p. i.

One of the critical factors that influence the power of detection of SNP (s)-associated with a trait is how much of the phenotypic variance is explained by the associated SNP (s). The highest the *h^2^* estimate, the chance of detecting SNPs increases. The *h^2^* estimates obtained in our study for the MAP load (log CFUs) within infected MDMs at 2 h and 7 d p. i. were 0.87 and 0.83, respectively. These findings suggest a strong influence of host genetics on MAP load within infected macrophages. To our knowledge, the *h^2^* estimate for the macrophages´ performance assessed by measuring MAP load within macrophages had not been estimated before and is higher than *h^2^* estimates calculated for other immunocompetence traits. For instance, the analysis of MDMs phenotypic variation revealed an *h^2^ =* 0.77 for the NO^-^ production by MDMs in response to *E.coli* (18). After performing the GWAS, we did not identify SNPs significantly associated with the NO^-^, Gal9, EREG, and C3 expression. However, six SNPs located on BTA2, BTA17, BTA18, and BTA21 were significantly associated with a lower MAP load within MDMs after 2 h p. i. This discordance can be explained because the MGIA is a functional assay that assesses the combined effects of a range of immune and metabolic mechanisms that influence mycobacteria growth *in vitro*, which is one of its advantages over measuring single predefined immune markers. It is important to highlight that for the assessment of viable intracellular MAP, we used the BACTEC MGIT 960 system, a fully automated solution for mycobacterial liquid culture that provides accurate measures of MAP load.

We identified six SNPs associated with low MAP load within MDMs in dairy cattle. Interestingly, the two identified SNPs located on BTA18 overlapped with QTLs associated with the somatic cell score (QTL:3556), length of productive life (QTL:3555), and reproductive traits such as stillbirth (QTL:11362), birth Index (QTL:30537), and dystocia (QTL:2707) highlighting the important role of this region (23.5-31.4 Mbp) on chromosome 18 in health, reproduction, and longevity (31–34, 58). This suggests that an animal that can limit MAP infection has also improved udder health, fewer reproductive problems, and, consequently, a longer productive life. In addition, the SNP located on BTA21 was located within QTLs associated with IgG levels (QTL:66212), somatic cell score (QTL:5446 and QTL:10190), and calving ease (QTL:11126) (35–38). This finding suggests that there might be genetic loci that impact both the primary innate immune response and the secondary production of IgG which is activated later if innate mechanisms are not able to eliminate the pathogen. Finally, the identified SNP on BTA17 was located within a QTL associated with calving ease (36). The identified SNPs on BTA17, BTA18, and BTA21 overlapped with QTLs for longevity, udder health, and reproductive traits. The identified markers could be useful for marker-assisted selection in dairy cattle breeding programs for PTB resistance in combination with other strategies like management and biosecurity.

Five candidate genes and one miRNA were identified 50,000 bp upstream and downstream of some of the identified SNPs; *Cysteine and Serine Rich Nuclear Protein 3* (*CSRNP3*), the *Coiled-Coil Domain Containing 92* (*CCDC92*), *Oxysterol Binding Protein like 6* (*OSBPL6*), *U6*, a non-characterized protein (ENSBTAG0000004285), and the bta-mir-2365. Specific functions of the four identified candidate genes are summarized in **Supplementary Table 2**. CSRNP3, also named TGF-Beta Induced Apoptosis Protein 2, is a transcriptional activator involved in positive apoptosis regulation. Apoptotic clearance of pathogens including MAP plays a critical function in the resolution of the infection by the removal of the infected macrophages along with the ingested organisms. CCDC92 is an interferon-γ stimulated protein that plays an important role in the regulation of innate immunity and attenuation of microbial infections (59). The OSBPL*6* is an intracellular lipid receptor that contributes to the maintenance of cholesterol homeostasis by regulating cellular cholesterol trafficking and efflux. Previously, genetic variants in candidate genes involved in cholesterol metabolism (bta04979) such as *Angiopoietin-like 4 and 8* (*ANGPTL4* and *ANGPTL8*), *Low Lipropotein Receptor* (*LDRL*), and *Neutral cholesterol Ester Hydrolase 1* (*NCEH1*) appeared enriched in animals with PTB-associated diffuse lesions (22). Consequently, reduced levels of plasma cholesterol were detected in cattle with diffuse lesions. Moreover, we observed that the cholesterol route (bta04977) was dysregulated in the ileocecal valve of cows with diffuse lesions with four upregulated *Apoliprotein* genes (*APOA1, APOC3, APOA4, APOB*) matching this route (54). Recent data suggest that MAP-infected macrophages accumulate intracellular cholesterol droplets and depict a foam phenotype during infection providing an enriched environment for MAP survival (60, 61). In addition to the observations that increased lipid biogenesis and transport to the place of the infection may favor MAP growth by the modulation of host immune response leading to an anti-inflammatory milieu, MAP may potentially mobilize lipid body content as a source of nutrients to enhance their survival and replication in host cells (62). In an opposite manner, our study suggests that animals with genetic variants in candidate genes favoring cholesterol homeostasis such as the *OSBPL6* might be able to limit MAP infection by reducing the lipid body content within infected MDMs. The *U6* spliceosomal RNA and the bta-miR-2365 were also identified as candidate genes in our study. The U6 is a small non-coding RNA involved in the post-transcriptional regulation of gene expression by affecting both the stability and translation of mRNAs. Using miRmap, we predicted target genes for the bta-miR-2365. When we used ClusterProfiler with the predicted target genes, an enrichment in genes associated with the endocytosis (bta04144) and the Hippo signaling pathway (bta04390) was found. The Hippo signaling pathway controls organ size by coordinately regulating cell growth, proliferation, and apoptosis (63). Emerging evidence has suggested that the innate immune response was extensively regulated by multiple core components of the Hippo cascade while the activation of innate immune signaling conversely led to substantial modulations of the Hippo pathway (64). In most cases, the Hippo signaling potentiates the innate immune response. In addition, the Hippo pathway-mediated processes are interconnected with those of other key signaling cascades, such as those mediated by TGF-beta and Wnt growth factors. Therefore, the bta-miR-2365 along with the other candidate genes identified in our study could modulate MAP’s entry and survival within macrophages. The candidate genes identified in our study were also compared to candidate genes previously identified in CD, IBD, and colorectal cancer to determine if there was any overlap. We found that *CSRNP3* was previously found to be associated with colorectal cancer (65). In clear cell renal cell carcinoma (ccRCC), the CSRNP3 may serve as a prognostic biomarker to predict the overall survival of patients (66). CSRNP3 might impact the immune environment of ccRCC through immunocyte infiltration of natural killer cells and plasmacytoid dendritic cells.

The sample size used in the current study could be considered small in comparison to traditional GWAS analysis using a larger population. However, the assessment of the macrophages´ performance by measuring MAP load within infected MDMs is a functional and controlled trait that can be measured in the absence of environmental and physiological effects. Other phenotypes, such as the NO^-^ response of bovine MDMs in response to *E.coli* or *S. aureus*, used approximately 60 samples to identify associated cis-SNPs (18). Further studies are needed to assess the capacity of macrophages to kill MAP as a phenotype for resistance to MAP infection in other cattle populations and ruminant breeds.

## 5 Conclusions

We report a new phenotype, the control of MAP early survival within infected macrophages, for evaluating resistance to MAP infection in dairy cows. This resistance is genetically determined and is mediated at the level of macrophages´ function. Considering how difficult it is to assess resistance to MAP infection, our approach represents a major advance in the field. The SNPs and candidate genes identified here revealed the association between MAP load within macrophages and genetics, which helps fill the knowledge gap between genetics, innate immune responses, and resistance to MAP infection in cattle. Our results define a heritable and distinct immunogenetic profile in MAP-infected macrophages associated with cholesterol homeostasis regulation and with the induction of apoptosis and IFNγ production. A strong effect of host genetics in limiting bacteria load within MDMs very early after infection was observed (*h^2^* estimate = 0.8) which opens the possibility of ranking Holstein cows based on predicted MAP survival variation within infected MDMs. The identified SNPs might be used to develop genetic evaluations for immunocompetence in the Spanish breeding program which would allow producers to select cattle more resistant to MAP infection and likely to other intracellular pathogens as well; ultimately reducing the prevalence of diseases and the dependence on antimicrobials, preventing economic losses, increasing the length of cattle productive life, and improving food safety.

## Data availability statement

The original contributions presented in the study are included in the article/**Supplementary Material**, further inquiries can be directed to the corresponding author.

## Ethics statement

The animal study was reviewed and approved by the Ethics Committee of Animal Experimentation of NEIKER.

## Author contributions

MC measured intracellular MAP load within MDMs, extracellular EREG, C3, Gal9, and NO^-^ levels, and performed the GWAS. GB-B performed the *gBLUP* model and contributed to the statistical analysis of the data. MC and GB-B performed the validation of the genomic predictions. MA-H was the principal investigator of the project and participated in project management, experimental design, and data analysis. All authors contributed to the article and approved the submitted version.
