# Supplementary figures and images for: Control of Mycobacterium avium subsp. paratuberculosis load within infected bovine monocyte-derived macrophages is associated with host genetics
Source: Front Immunol. 2023 Feb 22;14:1042638. doi: 10.3389/fimmu.2023.1042638 (PMC9992791; doi:10.3389/fimmu.2023.1042638)

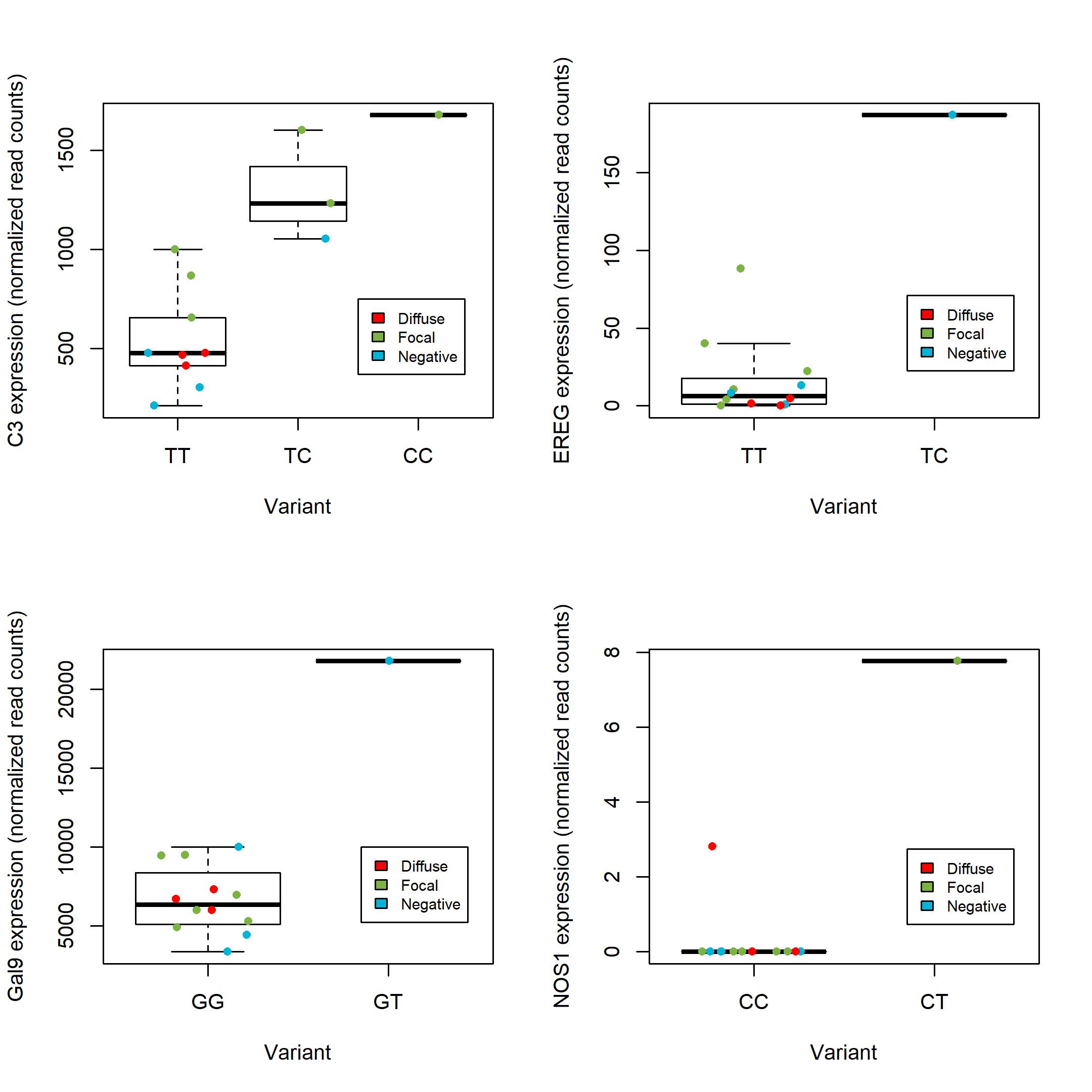

Supplement: Supplementary file 1 [file Image_1.jpeg]
