# Supplementary material for: Control of Mycobacterium avium subsp. paratuberculosis load within infected bovine monocyte-derived macrophages is associated with host genetics
Source: Front Immunol. 2023 Feb 22;14:1042638. doi: 10.3389/fimmu.2023.1042638 (PMC9992791; doi:10.3389/fimmu.2023.1042638)
Supplement: Supplementary file 3 [file Table_2.docx]

**Supplementary Table 2.** Specific functions of the four candidate genes identified in this study

| **Gene symbol** | **Function** |
| --- | --- |
| CSRNP3 | Binds to the consensus sequence 5'-AGAGTG-3' and has transcriptional activator activity |
|  | Plays a role in positive regulation of apoptotic process |
| U6 | required for the splicing process |
| OSBPL6 | Regulates cellular transport and efflux of cholesterol |
|  | Binds via its PH domain PI4P, phosphatidylinositol-4,5-diphosphate,  phosphatidylinositol-3,4,5-triphosphate, and phosphatidic acid |
|  | Weakly binds 25-hydroxycholesterol |
| CCDC92 | Interferon-stimulated protein that plays a role in innate immunity |
|  | Strongly inhibits ebolavirus transcription and replication. |
|  | Forms a complex with viral RNA-bound nucleocapsid (NP) and thereby prevents the transport of NP to the cell surface |
